# Molecular Pathways in Peritoneal Mesothelioma: A Minireview of New Insights

**DOI:** 10.3389/fonc.2022.823839

**Published:** 2022-02-10

**Authors:** Francesco Fortarezza, Federica Pezzuto, Andrea Marzullo, Domenica Cavone, Daniele Egidio Romano, Antonio d’Amati, Gabriella Serio, Luigi Vimercati

**Affiliations:** ^1^ Department of Cardiac, Thoracic, Vascular Sciences and Public Health, Pathology Unit, University of Padova, Padova, Italy; ^2^ Department of Emergency and Organ Transplantation, Pathology Unit, University of Bari, Bari, Italy; ^3^ Department of Interdisciplinary Medicine, Occupational Health Unit, University of Bari, Bari, Italy

**Keywords:** malignant mesothelioma, peritoneal mesothelioma, molecular pathways, prognostic factors, asbestos

## Abstract

Mesothelioma is a rare malignant neoplasm with poor survival. It mainly affects the pleura (90%) but can arise in all serous cavities: peritoneum (5-10%), pericardium and tunica vaginalis testis (<1%). The onset of pleural mesothelioma is strictly related to asbestos exposure with a long latency time. The causal link with asbestos has also been suggested for peritoneal mesothelioma, while the importance of exposure in the onset of pericardial and tunica vaginalis testis mesotheliomas is not well known. Mesothelioma remains an aggressive and fatal disease with a five-year mortality rate higher than 95%. However, new therapeutic approaches based on molecular-targeted and immunomodulatory therapies are being explored but have conflicting results. In this context, the identification of critical targets appears mandatory. Awareness of the molecular and physiological changes leading to the neoplastic degeneration of mesothelial cells and the identification of gene mutations, epigenetic alterations, gene expression profiles and altered pathways could be helpful for selecting targetable mechanisms and molecules. In this review, we aimed to report recent research in the last 20 years focusing on the molecular pathways and prognostic factors in peritoneal mesothelioma and their possible diagnostic and therapeutic implications.

## Introduction

Peritoneal mesothelioma (PM) is a rare malignant neoplasm that originates from the mesothelial cells lining the peritoneal serosa. PM was first reported in the early 1900s as a diffuse intraperitoneal neoplastic process associated with ascites in a young woman (1). The neoplasm represents 15-20% (2) of all mesotheliomas and shares some features with the most common pleural counterpart even if several substantial differences make it a separate and definite nosological entity. From an epidemiological point of view, PM more frequently affects females than males, with an earlier median age (52 years) (3) than pleural mesothelioma.

PM usually arises as multiple serosal nodules with thickening of the peritoneum. Adipose tissue invasion and stromal invasion represent indicative features of malignancy (4). Histologically, it is classified into three histotypes: a) epithelioid, with round monomorphic cells arranged into different architectural patterns; b) sarcomatoid, composed of spindle elements; and c) biphasic, with at least 10% of both components. Well-differentiated papillary mesothelioma is included among the mesothelial tumours of the peritoneum, which represents a rare variant with an indolent behaviour that occurs mainly in women of reproductive age (2, 5). The diagnostic algorithm mirrors that of the pleural diagnostic algorithm, with a combination of positivity for mesothelial markers and negativity for BAP1 (BRCA1 associated protein 1) in 40-60% of cases. The histologic variants are clinically relevant, allowing a prognostic stratification of patients and guiding the treatment strategies (6).

The link between asbestos exposure is weaker than that in pleural tumours. Even if asbestos exposure represents the most important risk factor (7), it is found in approximately 33-50% of cases compared to the frequency of over 90% in pleural mesothelioma. Furthermore, the latency period between asbestos exposure and the development of mesothelioma is 20 years for PM, compared to 30-40 years for pleural mesothelioma (8, 9). Asbestos fibres have been identified in the omentum and mesentery of the gastrointestinal tract (10). Various hypotheses have been formulated to explain the mechanisms by which asbestos fibres can reach the peritoneal cavity. A study showed significant incidences of pulmonary asbestosis (17%) and pleural plaques (26%) in a cohort of patients with PM, suggesting a link between pleuropulmonary and peritoneal diseases probably secondary to the migration of asbestos fibres (11). Another hypothesis concerns the migration of asbestos fibres through the female genital system from the uterus to the fallopian tubes up to the peritoneal cavity. Such contamination could take place following sexual intercourse or following the application of talc contaminated with asbestos, as described by some papers that have focused on mesotheliomas that originate from the germinative epithelium of the ovary, which represents a specialization of mesothelial cells (12). Other rare causes related to the onset of PM are those attributable to chronic inflammatory states of the peritoneal serosa, such as chronic peritonitis, recurrent Mediterranean fever, germline mutations of *BRCA* (BReast CAncer), endometriosis, the period following radiation therapy for other peritoneal or pelvic neoplastic processes, and simian vacuolating virus infection (13–15).

The strict association between asbestos exposure and the development of malignant mesothelioma is indisputable. Our understanding of the mechanisms of action of asbestos fibres and their effects on mesothelial cells has deepened since the middle of the twentieth century. Knowledge about carcinogenesis related to asbestos has increased together with awareness about the molecular changes in this tumour. Mesothelioma appears to be characterized by chromosome rearrangements and gene mutations/deletions (16). More recently, the molecular landscape of mesothelioma has been enriched by the discovery of susceptibility familial factors that influence the impact of asbestos, target mutations in oncogenes and tumour suppressor genes, and epigenetic changes (17, 18).

As treatment options for PM, as well as pleural PM, are currently limited and target therapies are far away from being available, a better understanding of the molecular pathogenesis could suggest further therapeutic opportunities. In this review, we aimed to report recent research in the last 20 years focusing on the most promising molecular pathways and prognostic factors in PM in terms of diagnostic and therapeutic implications.

## Discussion

A comprehensive review of the literature of the last decade was conducted in the Medline database, including research with the generic terms “peritoneal mesothelioma” AND “molecular”. All data were further confirmed by examining the list manually. The eligibility of studies was assessed by reading titles, abstracts, and full texts.

In **Tables 1** and **2** are respectively listed in chronological order the research studies and the case reports in which molecular analyses of PM were performed. Below, the most important molecular alterations that have emerged are discussed and grouped into “Oncogenes”, “Tumour suppressor genes” and “Post-transcriptional alterations” sections. Two additional sections are dedicated to the description of the role of the tumour microenvironment and on therapeutic approaches in PM.

**Table 1:** Previous molecular research studies.

Author	Main molecular alterations	Number of cases	Histotype	Technique	Type of samples
Foster JM et al. (19)	*EGFR*	29	NA	PCR	FFPE tumour
Perrone et al. (20)	*EGFR, PDGFRB, PDGFRA*	20	18E, 1B, 1S	PCR, FISH	FFPE tumour
Varghese et al. (21)	*PI3K and mTOR pathways*	41	E	PCR	FFPE tumour and non-tumoral tissue
Kalra et al. (22)	*EGFR*	33	E	PCR	FFPE tumour
Alakus et al. (23)	*BAP1*	7	E	WES	FFPE tumour and blood
Bozzi et al. (24)	*EZH2, c-MYC, E-cadherin, VEGFR2*	21	E	PCR, FISH	FF and FFPE tumour
Ugurluer et al. (25)	*BAP1, CDKN2A/B, NF2*	4	E	NGS	FFPE tumour
Desmeules et al. (26)	*EWSR1/FUS-ATF1*	1	E	NGS	FFPE tumour
Joseph et al. (27)	*BAP1, SETD2, DDX3X*	13	12E, 1B	NGS	FFPE tumour and non-tumoral tissue
Leblay et al. (28)	*BAP1*	46	41E, 3B, 2S	NGS	FF and FFPE tumour
Hung et al. (29)	*ALK, BAP1, NF2, SETD2*	9	7E, 2B	NGS	FFPE tumour
Serio et al. (30)	*DEF, UBE2Q1*	22	14E, 5B, 3S	Array-CGH	FFPE tumour
Panou et al. (31)	*BAP1, NF2, SETD2, PBRM1*	17	E	NGS	FFPE and tumour
Belfiore et al. (32)	*EGFR, HER2, HER3, Axl, MET, BAP1, NF2*	16	E	NGS	FFPE tumour and non-tumoral tissue
Shrestha et al. (33)	*BAP1, KANSL1, PBRM1, SETD2*	18	E	WES, WTS	FFPE tumour and non-tumoral tissue, blood
Sciarrillo et al. (34)	*SF3B1*	64	NA	IHC, RNAseq	TMA tumour, cell lines
Stevers et al. (35)	*TRAF7, CDC42*	10	WDPM	NGS	FFPE tumour and non-tumoral tissue
Brich et al. (36)	*BAP1, CDKN2A, NF2, SETD7, PCGF5*	75	71E, 2B, 2S	FISH, Array-CGH	FF/FFPE tumour, cell lines
Pezzuto et al. (37)	*WT-1, CDKN2A*	45	32E, 9B, 4S	IHC, FISH	FFPE tumour
Hung et al. (38)	*BAP1, ARID1B, PRDM1, PBRM1, SETD2, NF2, CDKN2A, P53, TRAF7, SUX12, ALK, CHECK2*, genomic near-haploidization	26	23E, 3B	NGS	FFPE tumour

B, biphasic; E, epithelioid; FF, fresh-frozen; FFPE, formalin-fixed paraffin-embedded; FISH, Fluorescent In Situ Hybridization; IHC, immunohistochemistry; NGS, next-generation sequencing; NA, not available; qRT-PCR, quantitative real-time polymerase chain reaction; PCR, polymerase chain reaction; RNAseq, REN-sequencing; S, sarcomatoid; TMA, tissue micro-array; WDPM, well differentiated papillary mesothelioma; WES, whole-exome sequencing; WGS, whole-genome sequencing; WTS, whole-transcriptome sequencing.

**Table 2:** Previous published case reports with molecular studies.

Author	Molecular alterations	Number of cases	Histotype	Technique	Type of samples
Hama et al. (39)	*KAZALD1, TMEM30B, MAPK13*	2	1E, 1S	DNA methylation analysis	FFPE tumour
Chao et al. (40)	*GNA11, JAK3*	1	E	NGS	FFPE tumour and blood
Sheffield et al. (41)	*NF2, CDKN2A, LATS2, MET*	2	B	WGS	FF/FFPE tumour and blood
Lai et al. (42)	*BAP1, NOTCH2, NSD1, PDE4DIP, ATP10B*	1	E	NGS	FF tumour and blood
Vanni et al. (43)	*WT1*	1	E	WES	FFPE tumour and blood
Loharamtaweethong et al. (44)	*STRN-ALK fusion*	1	E	FISH	FFPE tumour
Serio et al. (45)	Losses at 1q21, 2q11.1→q13, 8p23.1, 9p12→p11, 9q21.33→q33.1, 9q12→q21.33, and 17p12→p11.2	1	E	Array-CGH	FFPE tumour
Loffler et al. (46)	*PTEN*	1	E	NGS	NA
Lund-Andersen et al. (47)	*LATS1/2, BAP1, PBRM1*	1	NA	WES, RNAseq	FF tumour and blood
Rüschoff et al. (48)	*ALK*	1	E	NGS	FFPE tumour
Smith-Hannah et al. (49)	*VHL*	1	E	PCR	FFPE tumour
Glass et al. (50)	*NF2*	1	E	NGS	FFPE tumour
Miyagawa et al. (51)	*ALK*	1	E	NGS	FFPE tumour

Array CGH, Array comparative genomic hybridization; B, biphasic; E, epithelioid; FF, fresh-frozen; FFPE, formalin-fixed paraffin-embedded; FISH, Fluorescent In Situ Hybridization; NGS, next-generation sequencing; NA, not available; PCR, polymerase chain reaction; RNAseq, RNA-sequencing; S, sarcomatoid; WES, whole-exome sequencing; WGS, whole-genome sequencing.

### Oncogenes

Receptor tyrosine kinases are surface receptors that link growth factors, cytokines, and hormones, functioning as key regulators of normal cellular processes. Mutations in receptor tyrosine kinases alter signalling cascades, leading to dysregulation of protein expression.

*EGFR* has been investigated in the pathogenesis of PM. Foster et al. (19) found *EGFR* (Epidermal Growth Factor Receptor) mutations, both in L858R and other catalytic domains, in a high percentage of PM patients. In different studies, ligand-dependent activation (e.g., *HER2*- Human epidermal growth factor receptor 2, *HER3*, Axl, and *MET*-mesenchymal epithelial transition factor) and coactivation of *EGFR* and *PDGFRB* (Platelet Derived Growth Factor Receptor Beta) were shown, together with cooperation of these receptors with the mTOR (mammalian target of rapamycin) pathway, suggesting the potential efficacy of the combined inhibition of these cascades in PM (20, 32). In this direction, a patient with multicystic PM (52) and two patients with papillary PM (53) were successfully treated with rapamycin, an mTOR inhibitor. The mTOR signalling pathway closely interacts with *PI3K* (Phosphoinositide 3-kinases). Varghese et al. (21) detected the overexpression of genes in these pathways in patients with the shortest survival in a group of 41 PM patients treated with surgical cytoreduction and regional intraoperative chemotherapy perfusion. In subsequent studies, *EGFR* alterations emerged as more complex than somatic mutations, with the detection of silent polymorphisms and cooperation with other receptors, such as the formation of heterodimers, showing that PM do not harbour somatic mutations in the *EGFR* tyrosine kinase domain that would make them sensitive to molecularly targeted therapy (22).

In 2016, Loharamtaweethong et al. (44) described an anecdotic case of *ALK* (Anaplastic lymphoma kinase)-rearranged PM in a child, thus opening a new perspective in the molecular dissection of this entity. The presence of *ALK* alteration was the topic of investigation for the group of Hung et al. (29), who detected this new promising pathogenetic mechanism in 3% of patients, mostly young women. This epidemiologic distribution of *ALK* rearrangement was confirmed in more recent studies (38, 48, 51), all representing by striatin (*STRN*)-*ALK* fusion, an extremely rare *ALK* rearrangement reported in only 56 cancers of different organs (51). This evidence suggests the need to explore the alteration in such groups, for the possible use of molecular target therapies.

In the complex scenario of hidden alterations, some studies focused on alterations that could also be present in mesothelioma, translating the experience of other better characterized neoplasms. This is the case of *EWSR1* (EWS RNA Binding Protein 1) rearrangements. In 2013, Panagopoulos et al. (54) detected a specific fusion gene in mesothelioma. These results were further confirmed by Desmeules et al. (26), who associated *EWSR1* alteration with a unique subset of mesothelioma arising in young, nonexposed, *BAP1*-retaining patients, resulting in an undirect activation of c-*MET* gene.

*WT1* (Wilm’s tumor 1) gene mutations have been rarely described in PM, especially in those non-asbestos related (43). The immunohistochemical expression of WT1, which represents a useful diagnostic tool to assess the mesothelial origin of neoplastic cells, could also have a prognostic role associated with a better prognosis, as described by Pezzuto et al. (37).

### Tumour Suppressor Genes

Tumour suppressor genes regulate cell division and replication, thus leading to growth abnormalities when mutated, and their function is lost or reduced. This event seems to occur frequently in the development of PM. *BAP1* plays a key role in PM susceptibility and oncogenesis. Alakus et al. (23) described for the first time that the loss of *BAP1* occurred in PM in the absence of any other oncogenic drivers, such as *NF2* (neurofibromatosis type 2) and *CDKN2A* (Cyclin Dependent Kinase Inhibitor 2A). Although these genes have also been detected in the pleural form, some differences have been found between the two entities (25). This is mainly true for the prevalence of these alterations. In PM, a higher frequency of *BAP1* and a lower frequency of *CDKN2A* and *NF2* have been described (27, 55, 56). This could be related to the lower frequency in the peritoneum of biphasic and sarcomatoid mesothelioma that typically harbour these mutations (38). While *BAP1* was independent of the clinical outcome, the latter two have a negative prognostic significance, thus becoming interesting targets for therapeutic approaches. In contrast, Leblay et al. found that BAP1 protein nuclear expression mirrored molecular status, and its detection was a good and reliable prognostic marker for the complete loss of *BAP1* activity in PM (28). Particularly, the authors found a better overall survival for patients with *BAP1* mutations, protein expression loss, or at least one of these alterations independently of tumour histological subtype, age, and sex. In terms of biomarker discovery, Lai et al. (42) identified a tumour-specific neoantigen for *BAP1* following insertion of a frameshift mutation translated into a truncated protein which was predicted to be presented by the patient’s HLA-B molecule as a tumour-specific neo-antigen. A separate issue is represented by *BAP1* germline mutations. Together with somatic mutations, a *BAP1*-related cancer syndrome characterized by mesothelioma, uveal melanoma, and possibly other cancer types has been identified. This possibility should be taken into account when identifying patients at high risk. One group described a significant proportion of patients with mesothelioma carry germline mutations in cancer susceptibility genes, especially those with PM, absent asbestos exposure, second cancer diagnosis, and young age (31). As for the germline mutations of *NF2*, only very rare reports described the onset of a PM in the context of a type 2 neurofibromatosis (50). Two cases of PM have been described respectively associated with Cowden (46) and Li-Fraumeni syndrome (40). Von Hippel-Lindau (VHL) disease tumour suppressor gene *VHL* was found mutated in a unique case of clear cell epithelioid PM in a non-exposed women (49). Two tumour suppressor genes, *TRAF7* and *CDC42*, respectively involved in activation of mitogen‐ activated protein kinases (MAPKs) and in Rho GTPase signalling, were found mutually exclusively mutated in a series of well-differentiated papillary mesothelioma of the peritoneum (35), in absence of the typical mutations of the malignant counterpart, as those involving *BAP1*, *NF2*, *CDKN2A*, *ALK*, contributing to a clear-cut separation between the two entities. These last exceptional cases show how a specific molecular signature could correspond an unusual morphological variant and this should be kept in mind especially for diagnostic purposes to avoid misinterpretations.

### Post-Transcriptional Alterations

Although the genomics of PM has deepened, much less is known about the epigenomic landscape of this tumour. Starting from the evidence of the pleural form, epigenetic alterations in PM have also been suggested to contribute to carcinogenesis. Hama et al. (39) quantitatively analysed the methylation of *KAZALD1* (Kazal Type Serine Peptidase Inhibitor Domain 1), *TMEM308* (Tumor Microenvironment of Metastasis 308), and *MAPK13* (Mitogen-Activated Protein Kinase 13) and reported hypermethylation of these genes in PM. The authors found a correlation between *KAZALD1* and the sarcomatoid variant, showing a certain histotype distribution of molecular alterations. Bozzi et al. focused on epithelioid PM, where tumour cells were characterized by stemness and plasticity supported by epigenetic reprogramming in the context of mesenchymal epithelial reverse transcription. Thus, the authors suggesting that the PM is likely to be responsive to epigenetic regulator inhibitors, basing also on the inverse correlation between strong *EZH2* expression and the loss of the *BAP1* (24). One group studied splicing alterations in PM reporting an upregulation of spliceosomal genes with a high expression of *SF3B1* (Splicing factor 3B subunit 1) which correlated with a worse prognosis (34). An important posttranslational modification to target for the development of new therapeutic approaches for cancer treatment is ubiquitination. Recent studies have observed that ubiquitination is involved in the metabolic reprogramming of cancer cells. In a recent study, Serio et al. found several losses among which loss of function of ubiquitination and defensins in PM (30, 45, 57), suggesting an important role in the initial development and progression of neoplasia or in combination with other mechanisms.

### Role of the Tumour Immune Microenvironment (TME)

The interactions between tumour cells and immune cells are complex. This strict association is well recognized in several thoracic malignancies, such as lung cancer (58, 59). An increasing emphasis has been attributed to the immune milieu and thus to the employment of immunotherapy against mesothelioma. Most research has been conducted on the pleural form (60, 61), while much less is known about the characterization of the TME in the peritoneal form. It seems that the role of the TME is not detached from MM carcinogenesis. Shrestha et al. (33) found high checkpoint receptor activation in *BAP1* haploinsufficient PM, thus suggesting predictive value for tumour response to this marker. Similarly, White et al. (62) demonstrated a significant increase in PD-L1 (Programmed death-ligand 1) expression in PM patients with a high mutational burden and germline mutations.

## Advances in PM Treatment and Future Perspectives

Strictly connected to the topic of molecular alterations in PM is the development of new treatment strategies. To date, MM remains a rare cancer with only a few promising changes in treatment. This is mainly true for PM, as most of the knowledge is extrapolated from the pleural form. Together with the deeper awareness of the genomic alterations, therapeutic strategies targeting new pathways have been explored in the pleura with multiple trials available. As previously described, *BAP1* inactivating mutations are frequently detected, with itself or its multiple downstream pathways considered fascinating targets. In this scenario, *PARP* (63) and *EZH2* (Enhancer of zeste homolog 2) (64) inhibitors have been investigated in *BAP1*-negative tumours, with disappointing results. Similarly, *CDKN2A*, commonly deleted in mesothelioma with the loss of p16 protein expression, has been targeted in a clinical trial (65) with only limited success. Another well-known inactivation concerns *NF2*, leading to the loss of merlin protein and the dysregulation of several streams, among which the Hippo-YAP/TAZ pathways (66), potentially blocked by mTOR inhibitors (52, 53), warrant further analysis. An attractive target has also been found in mesothelin, a membrane glycoprotein that has been blocked in epithelioid pleural mesothelioma (67–69). Regarding rare alterations, anecdotal reports about *ALK* fusions in PM need to be reported in terms of the response to ALK inhibitors, such as in lung cancers (48). Immunotherapy deserves a separate discussion and is widely studied in MM, for which the best results have been obtained (70) with different administration strategies, alone or in combination, which represents a considerable goal.

## Conclusions

The molecular events that cause PM have not been clearly defined, and comprehensive genetic characterization remains challenging. Important steps have been made towards the definition of the molecular signature of neoplasia to identify therapeutic targets, along with the goals of other tumours. The recent published studies, particularly those based on next-generation or other high-throughput sequencing methodologies, show heterogenous molecular alterations, mostly involving *BAP1* and other DNA repair, chromatin, and cell cycle regulators. Awareness of the molecular and physiological changes leading to the neoplastic degeneration of mesothelial cells and the identification of gene mutations, epigenetic alterations, gene expression profiles and altered pathways could be helpful for selecting targetable mechanisms and molecules.

## Author Contributions

Conceptualization, FF, FP, GS, and LV. Methodology, AM and FP. Data curation DC, DR, and Ad’A. Writing—original draft preparation FF and FP. Writing—review and editing GS. Supervision, GS and LV. All authors have read and agreed to the published version of the manuscript.

## Conflict of Interest

The authors declare that the research was conducted in the absence of any commercial or financial relationships that could be construed as a potential conflict of interest.

## Publisher’s Note

All claims expressed in this article are solely those of the authors and do not necessarily represent those of their affiliated organizations, or those of the publisher, the editors and the reviewers. Any product that may be evaluated in this article, or claim that may be made by its manufacturer, is not guaranteed or endorsed by the publisher.
